# Peripheral Refraction of Two Myopia Control Contact Lens Models in a Young Myopic Population

**DOI:** 10.3390/ijerph20021258

**Published:** 2023-01-10

**Authors:** Maria Concepción Marcellán, Francisco J. Ávila, Jorge Ares, Laura Remón

**Affiliations:** Department of Applied Physics, University of Zaragoza, 50009 Zaragoza, Spain

**Keywords:** myopia control contact lenses, peripheral refraction, retinal eccentricity

## Abstract

Peripheral refraction can lead to the development of myopia. The aim of this study was to compare relative peripheral refraction (RPR) in the same cohort of uncorrected (WCL) and corrected eyes with two different soft contact lenses (CL) designed for myopia control, and to analyze RPR depending on the patient’s refraction. A total of 228 myopic eyes (114 healthy adult subjects) (−0.25 D to −10.00 D) were included. Open-field autorefraction was used to measure on- and off- axis refractions when uncorrected and corrected with the two CLs (dual focus (DF) and extended depth of focus (EDOF)). The RPR was measured every 10° out to 30° in a temporal-nasal orientation and analyzed as a component of the power vector (M). The average RPR for all subjects was hyperopic when WCL and when corrected with EDOF CL design, but changed to a myopic RPR when corrected with DF design. Significant differences were found between RPR curves with both CLs in all the eccentricities (Bonferroni correction *p* < 0.008, except 10°N). An incremental relationship between relative peripheral refraction at 30 degrees and myopia level was found. It is concluded that the two CLs work differently at the periphery in order to achieve myopia control.

## 1. Introduction

Myopia is defined as a refractive error where light rays entering the eye parallel to the optic axis are focused in front of the retina when accommodation is relaxed [1]. In general, this refractive error is characterized by increased axial length of the eyeball. The mean adult values for axial length range between 22–25 mm, and higher values are markers of axial myopia [2]. Pathological myopia can occur when the ocular axial length exceeds 26–27 mm (≥−5.00 D), which can be associated with serious ocular complications such as premature cataracts, glaucoma, and especially myopic macular degeneration and retinal detachment [3].

Currently, myopic population has increased in the last few years. In 2015, myopia was estimated to affect 27% of the global population. In 2020 the number of myopic patients reached 33% of prevalence. Holden et al. [4] predicted that 50% of the world population will be myopic in 2050 and that 9.8% will be affected by high myopia (i.e., pathological myopia). According to the published statistics, myopia is considered as a public health global problem and the associated economic burden is significant [5,6].

Moreover, myopia is a multifactorial condition resulting from the interaction between certain factors such as geographical, environmental, and genetic factors [7,8,9]. In Asia, the prevalence is approximately 50% higher than in Australia, Europe, and North and South America [6]. Children with one of the two parents presenting myopia have a probability of 21.4% to develop myopia, and genetics is also a risk factor for progressive myopia [9]. Children’s daily socio-cultural customs have drastically changed in the last decade. Spending less time outdoors directly translates into fewer hours of sunshine, and is thus is a risk factor for developing myopia [10] including the viewing of electronic devices [11]. In a recent clinical trial, it was shown that outdoor activities can inhibit progression in myopic children aged from 6 to 7 years by 30% in 1 year [12]. According to animal experiments [13,14], optical defocus at the peripheral retina is another factor that alters eye growth, with specifically hyperopic peripheral defocus resulting in an increase in the axial length. In addition, some studies proposed to analyze the interaction between peripheral defocus and high-order aberrations for the development of myopia [15,16,17].

To prevent the risk of eye disease caused by myopia, it is crucial to control its progression. In that sense, various treatments were developed to control the progression of myopia such as pharmacological options and optical aids (contact lenses and ophthalmic lenses). Regarding contact lenses (CL) for myopia control, a great number of specific CL geometries based on two principles were proposed. In the first design, an induced peripheral myopic defocus was created, showing its effectiveness in slowing the growth of axial length [18,19,20,21,22]. Specifically, soft peripheral gradient soft CLs [23], aspheric centre-designs multifocal [24], and orthokeratology [25] were proposed to induce changes in peripheral refraction. The second design principle used extended depth of focus CL that were designed to obtain a stable retinal image quality that was improved for points on and anterior to the retina and degraded for points posterior to the retina to control axial elongation [21]. Currently, two CLs for myopia control are marketed under the name of MiSight (Coopervision), based on the principal design to induce peripheral myopic defocus and Mylo (mark’ennovy), based on the principal design of extended depth of focus (EDOF).

Then, considering the importance of peripheral retina refraction in myopia control, this study aims to investigate and compare the alterations of relative peripheral refraction (RPR) during wearing two different soft CLs types designed for myopia control: dual focus (DF) myopia control (MiSight) and extended depth of focus CLs (Mylo). In addition, the peripheral refractive errors are analyzed depending on the patient’s refraction (PR). This is a very important question in myopia control, i.e., patients with different refractive degrees have different degrees of peripheral hyperopic defocus.

## 2. Materials and Methods

### 2.1. Patients

This study was approved by the Ethics Committee for Clinical Research of Aragon (PI20/377) and adhered to the tenets of the Declaration of Helsinki. All participants were informed about the nature, risks, and possible adverse consequences of the study and signed an informed consent document.

The participants were European Caucasian subjects and non-wearers of contact lens students from the school of Optics and Optometry of the University of Zaragoza (Spain). A total of 228 eyes from 114 healthy young adult subjects (mean age of 23.11 ± 0.36 years old) were involved in the study.

Inclusion criteria for the study were an age between 18 and 29 years old, a myopic refractive error between −0.25 D to −10.00 D, refractive astigmatism of −0.75 D or below, and corrected distance visual acuity with the spherical equivalent (SE) of 0.8 decimal or better. Exclusion criteria were a history of previous ocular and systemic pathologies; having undergone refractive surgery; using medications that produce variations in accommodation, pupil size, tear film or refraction; amblyopia; and strabismus.

### 2.2. Contact Lenses

Two types of contact lens were used in this study. Both CLs were specially designed to control the progression of myopia.

The MiSight 1 Day soft contact lens (Coopervision, San Ramon, CA, USA) is a hydrogel (Omafilcon A) daily replacement option. The material has a high-water content (60%) and is manufactured with a single 8.70 mm base curve, a diameter of 14.20 mm, and negative optical power (−0.25 to −10.00 D). This CL utilizes a dual-focus concentric ring design with alternating vision correction zones and treatment zones. The center zone of the lens corrects the wearer’s vision. The treatment zones add +2.00 D to the distance power correction providing peripheral myopic defocus.

The Mylo (Mark’ennovy Personalized Care SL, Majadahonda, Madrid, Spain) has an extended depth of focus CL. This CL is manufactured in silicone hydrogel (Filcon A) for monthly replacement. The material has a high-water content (75%) and is manufactured with base curves from 7.10 to 9.80 mm, diameter (13.50–15.50 mm), and negative optical power (−0.25 to −15.00 D). From the optical design, it is based on the use of a distribution of power from the centre to the periphery, which is not progressive, especially in the optic zone (i.e., there were no discrete power zones and the power varied above and below the normal mean power). The peripheral optic zone leads to a controlled induction of higher order aberrations to achieve a thorough global focus (both central and peripheral) retinal image quality that was optimized for points at and anterior to the retina and degraded for points posterior to the retina, allowing enlargement of the depth of focus.

### 2.3. Clinical Protocol

The measurements were realized in the Optometric External Care Practice (Faculty of Sciences, University of Zaragoza) under the same experimental lighting conditions. The pupil size and accommodation were not controlled artificially. The examination room illumination was dimmed (mean of three measurements: 70 ± 8 lux, measured using a PCE-174 Luxmeter) to obtain a pupil size sufficiently large enough to measure the peripheral retina without using dilation drops. All measurements were conducted by three experienced optometrists.

The study consisted of three visits. In the first visit, the spherical equivalent (SE) refractive for each subject was determined using a non-cycloplegic objective (Grand-Seiko WAM-5500, Grand-Seiko Co., Ltd., Hiroshima, Japan) as well as subjective refraction following the standard procedure, until the maximum positive value was reached, thus providing the maximum visual acuity and Jackson cross-cylinder technique. The visual acuity evaluation was measured monocularly with the SE at a distance of 6 m using 24 inches of a commercial digital display with Snellen optotypes controlled by OptoTab(R) (SMTV Researching SL, Zaragoza, Spain) [https://smarthings4vision.com/, accessed on 28 November 2022]. Biomicroscopic anterior eye segment examination by means of slit lamp (BQ 900, Haag-Streit International) was performed. The first trial CL was chosen using the following criteria: (1) the total diameter was adjusted depending on the horizontal visible iris diameter for the Mylo CL, (2) the back vertex power was closer to the spectacle refraction after correction of vertex distance power, and (3) the back optic zone radius was chosen following the manufacturer’s guidelines. Non-cycloplegic central and relative peripheral refractions were measured using an open-view autorefractometer (Grand-Seiko WAM-5500). The tracking targets were located at 2.28 m arranged horizontally at the positions corresponding to the eccentricities from −30° temporal visual field (temporal retinal area) to 30° nasal visual field (nasal retinal area), in 10° steps. Fixation targets were red circles located on a wall (whose sizes corresponded to a visual acuity of 0.3 logMAR). In total, 7 positions representing the 60° of the visual field were measured; 6 measurements in the peripheral retina; and a central measurement coinciding with the fovea. Measurements were taken with the eye rotation technique. In each sight position, three refraction measurements were taken at each target fixation and the average was obtained.

In the second and third visit, the CLs testing was performed. The wearing sequence for the two CL was randomly assigned, and at least one week was left between treatments, ranging from 7 to 10 days. The visual acuity was measured, and a slit-lamp examination was performed to assess a correct lens fitting. After 1 h of wearing, the central and relative peripheral refractions were measured following the same procedure described above at the first visit.

### 2.4. Data Analysis

Refractions in clinical notation (sphere, cylinder, and axis) were converted to vector components [26]: spherical equivalent M, with/against the rule astigmatism, J_0_; and oblique astigmatism, J_45_. Only the M component was considered in this study. For each condition (baseline, DF (MiSight) and EDOF (Mylo)), relative peripheral refractive error for the M value was calculated for each eccentricity as the difference between peripheral refraction and central refraction as RPR = M_Exc_ − M_0_. Negative values correspond to the temporal visual field (TVF)/temporal retina, and positive values to the nasal visual field (NVF)/nasal retina.

The data of the RPR without contact lens (WCL) were analyzed depending on the patient’s refraction with two different and complementary methods. The first method consisted of performing linear regression analyses (patient’s refraction (PR) vs. RPR) for the most extreme eccentricities (30 degrees nasal and 30 degrees temporal). In a second method, four refractive groups according to subjective SE refractive error were made: very low myopia group (0.25 D to −0.75 D), low myopia group (−1.00 D to −2.25 D), moderate myopia group (−2.50 D to −3.75 D), and high myopia group (−4.00 D to −9.50 D). They were previously made to perform multiple comparisons of all possible pairwise population means.

The statistical analysis was performed using SPSS statistics software (SPSS Inc, Chicago, IL, USA). The normality of all sets of data was analyzed with Shapiro-Wilk tests; nonparametric tests (Krushal-Wallis H-test, Wilcoxon, and U-Mann) were performed when data normality was discarded. In particular, the Wilcoxon test was used for the comparisons between pairs since the U-Mann test was used to compare the RPR curves of both CL designs for each eccentricity of the visual field. The level of significance was set at 5%. Any multiple comparison was adjusted using Bonferroni correction (*p* < 0.008). Post hoc power (1 − β) and effect size (d) calculations were performed in cases where the null hypothesis was not rejected. The data were grouped according to their effect size, d = 0.2 small, d = 0.5 medium, and d = 0.80 large. The expected statistical power for an analysis is 80%.

## 3. Results

### 3.1. Descriptive Characteristics

A total of 228 eyes from 114 healthy young adult subjects (mean age of 23.11 ± 0.36 years old, range between 18 and 29 years) were involved in the study. There were 18 men and 210 women. The average refraction, expressed in SE, was −2.78 ± 1.78 D with a range between −0.25 D and −9.50 D. Table 1 shows the distribution of refractive groups according to subjective SE. For the distance visual acuity monocularly with the SE, the average baseline value was −0.04 ± 0.05 logMAR. When wearing the study lenses, the average values were −0.06 ± 0.08 logMAR for DF and 0.02 ± 0.08 logMAR for EDOF.

### 3.2. Relationship between RPR and PR without CL

#### 3.2.1. RPR Linear Regression at Extreme Eccentricities with All the Population

The linear regression analysis (RPR = bxPR + a) for 30 degrees temporal field and 30 degrees nasal field with all the population included and resulted, respectively, in b_30T = −0.11 ± 0.05 (*p* = 0.0482), a_30T = +0.68 ± 0.18 D (*p* = 0.0002), R-squared = 0.025; and b_30N = −0.13 ± 0.06 (*p* = 0.0276), a_30N = +0.92 ± 0.19 D (*p* < 0.0001), R-squared = 0.030. Slope (b) and intercept coefficient (a) differences between both extreme fields were found not statistically significant. Averaging both slope and intercept coefficients results in b_30TN = −0.12 and a_30TN = +0.80 D average values.

#### 3.2.2. RPR at Different Eccentricities among Refractive Groups

**Figure 1:**
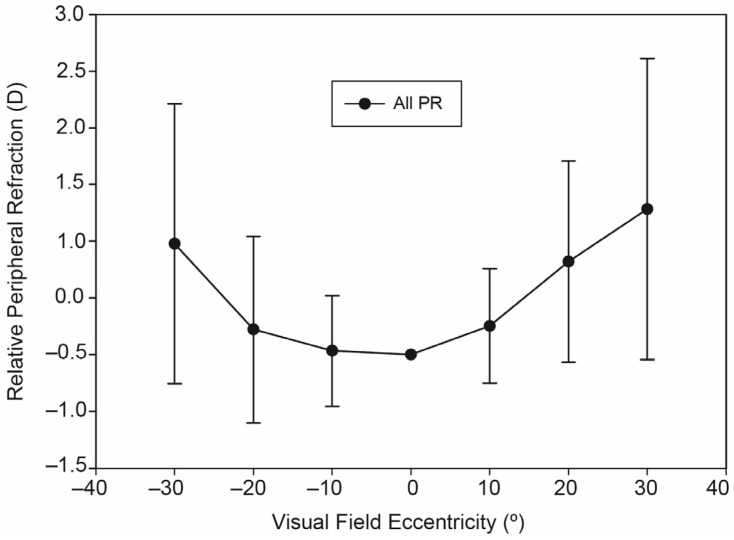
Average values and standard deviation of RPR without CL for all data samples.

Figure 1 represents the mean and standard deviation of RPR without CL for all data samples (see also Table 2, first line). It is shown that the RPR increased with increasing eccentricity and WCL provided relative peripheral hypermetropic defocus values for all eccentricities, which went up to a maximum value of +1.28 ±1.33 D at the 30°-degree nasal location. At the −30° degree temporal location this value is equal to +0.98 ±1.23 D. Mean peripheral refraction was less hyperopic for the temporal than for the nasal field, although this difference is not statistically significant (*p* = 0.091).

**Figure 2:**
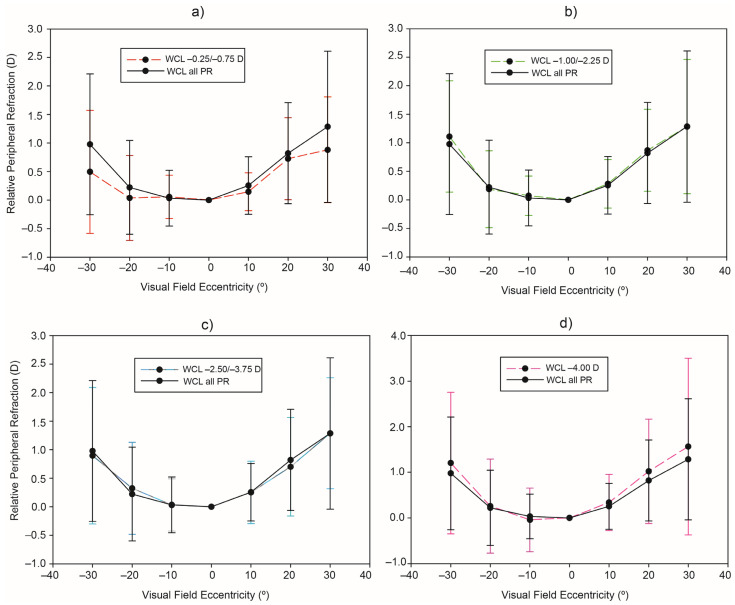
Average values and standard deviation of RPR without CL as a function of the patient’s refraction. Comparison between the mean of the RPR ensemble and (**a**) very low refraction, (**b**) low myopia refraction, (**c**) moderate myopia refraction, and (**d**) high myopia refraction.

Figure 2 shows the mean and standard deviation of RPR without CL as a function of the patient’s refraction (data are shown also in Table 2). At an eccentricity of −30° TVF, the RPR in the four refractive groups were: very low myopia group, +0.49 ± 1.08 D; low myopia group, +1.11 ± 0.98 D; moderate myopia group, 0.89 ± 1.20 D; and high myopia group, +1.20 D ± 1.55 D. At an eccentricity of 30° NVF, the RPR in the four refractive groups were: very low myopia group, 0.88 ± 0.93 D; low myopia group, +1.28 ± 1.18 D; moderate myopia group, +1.29 ± 0.97 D; and high myopia group, +1.56 ± 1.94 D. The mean RPR of the very low myopia group was smaller than that of the other refractive groups at the eccentricity of 30° TVF and NVF. However, there were not statistically significant RPR differences between patient’s refraction groups for all eccentricities (see Table 2, last line).

### 3.3. RPR at Different Eccentricities with DF and EDOF Design

**Figure 3:**
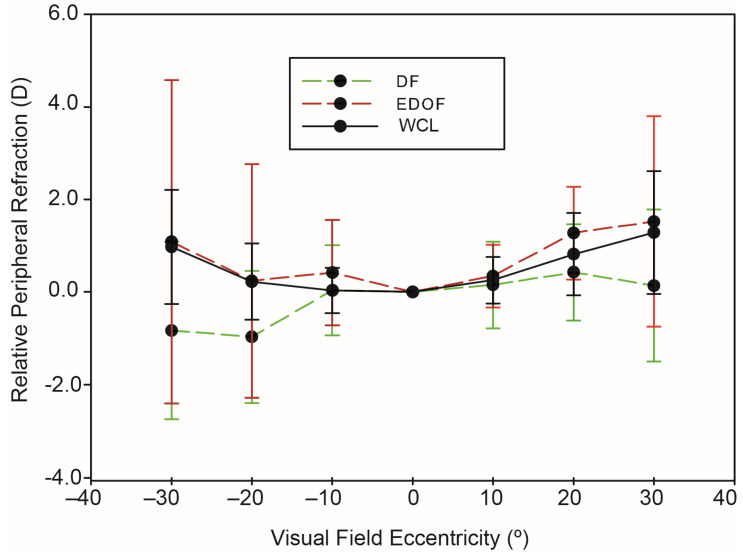
Average values and standard deviation of RPR measured as without CL and with DF and EDOF contact lenses.

Figure 3 shows the peripheral refraction curves measured with WCL and with the DF and EDOF contact lenses. Table 3 shows the data represented in Figure 3. Compared to the central refraction, the DF contact lens produced a relative peripheral myopic defocus at each position (green dashed line). At eccentricities between 20° and 30° at the TVF, RPR values were −0.98 ± 1.42 and −0.83 ± 1.91 D, respectively. At an eccentricity of −30° TVF, there is a −1.81 D myopic shift difference compared to the WCL (solid black line). At an eccentricity of 30° NVF, there is a −1.14 D myopic shift difference compared to the WCL condition. Compared to the central refraction, the EDOF contact lens produced a relative peripheral hyperopic defocus at each position, which went up to a maximum value of +1.56 ± 2.30 D at the 30° nasal visual field. The relative peripheral refraction curves measured with the EDOF lens (red dashed line) behave similarly to mean peripheral refraction WCL. In fact, unlike DF, a small hyperopic shift can be observed for EDOF lenses.

Table 4 shows the Wilcoxon signed rank test of RPR at different visual field eccentricities for DF lenses versus no CL and EDOF lenses versus no CL. Compared to the RPR without CL and with the DF contact lenses, there were statistically significant differences for the eccentricities of 30 T, 20 T, and 30 N (with significant differences between both curves). However, the peripheral positions of the visual field corresponding to 10 T, 10 N, and 20 N there was no statistical significance. The DF lens produces a myopic shift in the peripheral retina, although an RPR of less than −0.25 D can be observed for eccentricities of −10°T and 10°N. Compared to the RPR without CL and with the EDOF contact lens, a hypermetropic shift was produced, with no significant correlations in eccentricities of −30 T, −20 T, 10 N, and 30 N. Statistical differences are found at 10 N and 20 T, producing greater hyperopia at these points than with CL.

Table 5 shows the U-Mann test in order to study whether the RPR curves of the DF design and the EDOF match the different eccentricities of the VF. The RPF curves are compared, they show significant differences in all the eccentricities except 10°N.

## 4. Discussion

There is evidence of peripheral refraction’s role in myopia development. The Smith [27] work with monkeys indicates that the peripheral retina is important to emmetropisation and myopia development. Optical defocus at the peripheral retina alters eye growth, with specifically hyperopic peripheral defocus resulting in an increase in the axial length [14,28,29]. However, other authors [30] show that peripheral hyperopic shift is not related to the development of axial myopia. For that, the relationship between RPR and myopia remains controversial and the mechanism by which the peripheral defocus affects axial length growth requires further studies.

In the present study, we investigate and compare the alterations in RPR wearing two different CL types designed for myopia control: dual focus myopia control (MiSight) and extended depth of focus CLs (Mylo). Moreover, the peripheral refractive errors are analyzed depending on the patient’s refraction (PR), i.e., to what extent patients with different refractive degrees have different degrees of peripheral hyperopic defocus.

Our results show that the RPR without CL in myopic patients (all samples) provided a significant hyperopic shift at the peripheral retina (see Figure 1). The magnitude of the shift was comparable with that previously reported in other studies [31,32,33,34]. Moreover, as can be seen in Section 3.2.1, a statistically significant linear increasing trend (*p* < 0.05) was found for RPR measured at 30 degree eccentricities (nasal and temporal) in the function of myopia degree (decreasing when the sign of refraction is included). On the one hand, according to the average slope results for both extreme eccentricities an increase of −1.00 D of myopia degree should increase the average RPR by +0.12 D. On the other hand and according to the averaged zero intercept coefficients, +0.80 D average hyperopic RPR must be expected for emmetropic patients (PR = 0). In close relation to this, Mutti et al. [35] found that each dioptre of hyperopic RPR conferred a greater risk of the onset of myopia and that it depends on the ethnic group. It was reported [36] that Asian adults had steeper retinas and greater hyperopic shifts than in Caucasian adults. Myopes had steeper retinas and greater relative hyperopic peripheral refractions than emmetropes.

The findings of the BLINK study showed that greater foveal myopia was associated with more relative peripheral hyperopia and shorter peripheral eye lengths [31]. In that study, a total of 294 children aged 7 to 11 years with −0.75 to −5.00 D of myopia were considered. Conversely, our analysis regarding different myopic groups (Section 3.2.2) does not show any significant difference between peripheral refractive errors and the prescription refraction (see Figure 2). Similar results were found by Furuse et al. [33]. They did not find evidence for the hypothesis that greater myopia and longer axial length are associated with a greater peripheral hyperopic shift of the refraction. The apparent contradiction between 3.2.1 and 3.2.2 (and some published studies) results leads us to think about the relevance of the analysis methodology to explore the relationship between RPR and PR. Closely related to this, it is important to note that Mutti et al. [37] found that the fastest rate of change in refractive error, axial length, and relative peripheral refractive error was during the year before onset rather than in any year after onset.

Analysis of peripheral refraction patterns when wearing different contact lenses was reported in the literature. It was described recently that under-correction, full correction, and overcorrection of central refractive error with single vision soft CL causes hyperopic shifts in the peripheral visual field [38]. On the contrary, Shen et al. [39], using spherical CLs, reported the ability to decrease the amount of relative peripheral hyperopia. However, other studies reported that single vision CLs do not produce an effect on the change in the rate progression in myopia in a test group when compared with a control group wearing spectacle lenses [40,41]. De la jara et al. [42] concluded that the power profile across the optic zone of CL has an influence on the peripheral refractive error of the eye. Overnight Ortho-K CL wear shifted the RPR in the myopic direction in comparison with single vision spectacles and soft contact lenses [25]. More robust evidence for slowing myopia was found with the use of multifocal center-distance contact lenses that induce myopic peripheral defocus, which may be inhibitory for axial elongation. Several studies showed that wearing multifocal contact lenses can reduce myopia progression compared to wearing single CL [43,44].

A great number of specific contact lens (CL) geometries were designed to control the progression of myopia [18,19,21,22]. Different studies on the evaluation of CLs for myopia control are focused on determining their efficacy in decreasing the progression of myopic refractive error as well as reducing axial elongation [21,45]. Recently, Chamberlain et al. [45] concluded that the eye growth of the initial control group with DF contact lenses was slowed by 71% over the subsequent 3-year treatment period. Sankaridurg et al. [21] found a reduction in SE from 24% to 32% with the EDOF lenses.

Our results show that the RPR at different eccentricities with the DF contact lenses produced a relative peripheral myopic defocus at each position compared to the central refraction (see Figure 3 and Table 3). Comparing the WCL curve with the DF curve can be observed a myopic shift at an eccentricity of 30° of −1.81 D for the temporal retina and −1.14 D for the nasal retina. We found at the eccentricities of 30 T, 20 T, and 30 N there is a statistically significant difference between RPR without CL and with the DF contact lens. However, in peripheral positions of the visual field corresponding to 10 T, 10 N, and 20 N there was no statistical significance (see Table 4). Ji et al. [46] modelled single-vision, bifocal, and DF lenses using a ray tracing program based on their power maps. They found that the DF lens at eccentricities of 30° showed a myopic shift of −0.75 D, while the bifocal showed a hyperopic shift of +0.20 D. Berntsen et al. [47] analyzed peripheral defocus when myopic eyes are corrected with spherical and centre-distance multifocal soft contact lenses. At distance, the multifocal contact lenses resulted in significantly more myopic defocus than the spherical contact lenses at the 40° and 30° locations on the nasal retina, and at the 20° and 30° locations on the temporal retina. In that study, they used a centre-distance multifocal soft contact lens with a +2.50-D add (Biofinity Multifocal “D”) and in the present study, we have used a DF design (Misight) that adds +2.00 D to the distance power correction providing peripheral myopic defocus. Despite this difference, the designs of both lenses are very similar. They concluded that the defocus profiles experienced with the multifocal contact lens are suitable for studies seeking to examine the effect of peripheral myopic defocus on myopia progression in children.

Unlike what happens with the DF contact lens, we found a hyperopic shift for the EDOF contact lenses. Compared to the central refraction, EDOF contact lens produced a relative peripheral hyperopic defocus at each position (see Figure 3 and Table 3). Compared the RPR without CL and with the EDOF contact lens, a hypermetropic shift was produced, with no significant difference in eccentricities of 30 T, 20 T, 10 N, and 30 N. However, statistical differences were found at 10 N and 20 T (see Table 4). Sankaridurg et al. [21] developed a study with an extended depth of focus (EDOF), i.e., Mylos prototype and found that the power profile of these novel lenses varied across the entire optical zone with no discrete zone constant power. Although the RPR curve of our study does not match with that of the Sankaridurg study, in both cases when compared to the WCL curve, it can be seen that they are more hyperopic than with the study lens.

The RPR curves for both types of lens have different behaviours, i.e., the EDOF lenses produces a hyperopic shift in the peripheral retina instead of a myopic shift as occurs with the DF. We found that there is a significant difference in all the eccentricities except 10 °N (see Table 5). However, as both contact lenses are supported by studies to control the progression of myopia [21,45] and taking into account the particular peripheral geometry of the EDOF CL [21], it might be speculated that the bad peripheral image quality is what produces the myopia control effect in the EDOF CL.

Finally, we found an asymmetry between the eccentricity of 30° nasal and −30° temporal for WCL, although the difference was not statistically significant (*p* = 0.091). Mutti et al. [31] also found negligible nasal and temporal asymmetry in Caucasian patients. Kang et al. [48] found differences between moderately myopic East Asians and Caucasians, the Asians presenting higher hyperopic defocus in the peripheral retina and nasal-temporal asymmetry.

Nevertheless, this study was not devoid of limitations. Firstly, because of the technique used to measure peripheral refraction, the maximum peripheral eccentricity to measure RPR was 30 degrees. This limitation contrasts with some other studies where higher eccentricities were explored [49]. Furthermore, it is considered that 30 degrees is a good reference value to compare with other studies [35,50] about myopia control with contact lenses. Second, as soft contact lens shape can be modified during wearing use by dehydration [51], it can be argued that our RPR is only a snapshot of the peripheral refraction (1 h wearing use) which is being experienced by the users throughout the day. More studies should be done to analyse how the RPR is changing during wearing time. Third, central and peripheral refraction were carried out without cycloplegia in order to measure them in the most natural condition. As a result, despite the fact that non-accommodative stimuli and an open-field auto-refractometer were used to minimize the accommodative response of our patients, it can be argued that our measurements might be affected by some spurious accommodative “myopic” noise. However, because all peripheral refractions are relative (by means of subtraction) to the central ones, it is expected that the influence of this effect is negligible. In relation to the research between RPR and PR, future research may consider topography, higher-order aberration, corneal biomechanics, and axial length as class variables. In addition, larger eccentricities may consider to characterise the peripheral retina. Finally, only VA was measured as an indicator of visual quality so other subjective parameters such as contrast sensitivity, stereoacuity, or perception of halos could be considered in future studies.

## 5. Conclusions

The power profile across the optic zone of CL has an influence on the peripheral refractive error of the eye. Relative peripheral refractions showed myopic defocus with the dual focus (Misight) contact lenses. Correcting the same eyes with extended depth of focus (Mylo) contact lenses results in hyperopic defocus in the peripheral retina. However, previous studies are focused on determining the efficacy in decreasing the progression of myopic refractive error as well as reducing axial elongation for the two different myopia control CL designs. Our results show that the RPR without CL in myopic patients provided a significant hyperopic shift at the peripheral retina. Finally, the apparent contradiction in the statistical significance of the relationship between RPR and PR when different methodologies were used, suggests that further studies must be conducted to address this relevant subject.

## Figures and Tables

**Table 1:** Distribution of refractive groups according to subjective SE.

Number of Eyes	Subjective SE (D)
34	−0.25/−0.75
73	−1.00/−2.25
71	−2.50/−3.75
50	−4.00/−9.50

**Table 2:** Relative peripheral defocus M data WCL for all data samples (first line) and as a function of the patient’s refraction. Each cell displays the mean and standard deviation. Kruskal-Wallis *p*-value of the RPR at different eccentricities of the visual field. Effect size 1 = 0.2 small, 2 = 0.5 medium, and 3 = 0.80 large. P = 1 − β ≥ 80%.

Eccentricity		30 T	20 T	10 T	0	10 N	20 N	30 N
WCL	All PR	+0.98 ± 1.23	+0.22 ± 0.82	+0.03 ± 0.49	0	+0.25 ± 0.50	+0.82 ± 0.89	+1.28 ± 1.33
WCL	−0.25/−0.75 D	+0.49 ±1.08 ^3P^	+0.04 ± 0.74 ^3P^	+0.06 ± 0.38 ^1^	0	+0.14 ± 0.33 ^3P^	+0.72 ± 0.72 ^3P^	+0.88 ± 0.93 ^3P^
WCL	−1.00/−2.25 D	+1.11 ± 0.98 ^1^	+0.19 ± 0.68 ^1^	+0.07 ± 0.35 ^1^	0	+0.28 ± 0.43 ^3P^	+0.87 ± 0.72 ^1^	+1.28 ± 1.18 ^1^
WCL	−2.50/−3.75 D	+0.89 ± 1.20 ^2P^	+0.32 ± 0.80 ^2P^	+0.03 ± 0.46 ^1^	0	+0.25 ± 0.55 ^1^	+0.70 ± 0.86 ^2P^	+1.29 ± 0.97 ^1^
WCL	−4.00/−9.50 D	+1.20 ± 1.55 ^3P^	+0.26 ± 1.03 ^1^	+0.04 ± 0.69 ^1^	0	+0.34 ± 0.62 ^2P^	+1.02 ± 1.14 ^3P^	+1.56 ± 1.94 ^3P^
*p*-value		0.352	0.539	0.691		0.428	0.514	0.145

**Table 3:** Relative peripheral defocus M data for WCL, EDOF, and DF contact lenses. Effect size 1 = 0.2 small, 2 = 0.5 medium, and 3 = 0.80 large. P = 1 − β ≥ 80%.

Eccentricity	30 T	20 T	10 T	0	10 N	20 N	30 N
WCL (D)	+0.98 ± 1.23	+0.22 ± 0.82	+0.03 ± 0.49	0	+0.25 ± 0.50	+0.82 ± 0.89	+1.28 ± 1.33
EDOF(D)	+1.09 ± 3.54 ^3P^	+0.30 ± 2.54 ^1^	+0.40 ± 1.14	0	+0.36 ± 0.68 ^1^	+1.25 ± 0.99	+1.56 ± 2.30 ^1^
DF(D)	−0.83 ± 1.91	−0.97 ± 1.42	+0.04 ± 0.97 ^1^	0	+0.15 ± 0.93 ^1^	+0.42 ± 1.04 ^2P^	+0.14 ± 1.64

**Table 4:** Wilcoxon signed-rank test of RPR at different visual field eccentricities for EDOF lenses versus no CL and DF lenses versus no CL. Asterisks indicate significant differences with Bonferroni correction.

*p*-Value	30 T	20 T	10 T	10 N	20 N	30 N
EDOF	0.129	0.50	0.000 *	0.366	0.000 *	0.010
DF	0.000 *	0.000 *	0.985	0.180	0.625	0.001 *

**Table 5:** U-MANN of RPR at different visual field eccentricities comparing the DF curve versus the EDOF curve. Asterisks indicate significant correlations with Bonferroni correction. P = (1 − β) > 80%.

	30 T	20 T	10 T	10 N	20 N	30 N
*p*-value	0.000 *	0.000 *	0.001 *	0.229 ^P^	0.000 *	0.000 *

## Data Availability

The data presented in this study are available on request from the corresponding author. The data are not publicly available due to privacy.
